# Unintended Pregnancy and Associated Factors among Pregnant Women Attending Antenatal Care at Bako Tibe District Public Health Facility, Oromia Region, Ethiopia

**DOI:** 10.1155/2020/3179193

**Published:** 2020-03-19

**Authors:** Habtamu Bekele, Merga Dheressa, Bezatu Mengistie, Yitagesu Sintayehu, Gelana Fekadu

**Affiliations:** ^1^School of Nursing and Midwifery, College of Health and Medical Science, Haramaya University, Ethiopia P.O. Box: 235; ^2^Department of Environmental Health, College of Health and Medical Science, Haramaya University, Ethiopia P.O. Box: 235

## Abstract

**Background:**

A pregnancy is described unintended if it is either unwanted or mistimed. The former occurs when no child or no more children are desired, and the latter is when the conception occurs earlier than the desired time, but wanted later. Unwanted pregnancy causes a serious health, economic, and social problem to the woman and her family. In the study area, there is limited data on unintended pregnancy. Therefore, this study fills this gap by studying the magnitude of unintended pregnancy and its associated factors among pregnant women attending antenatal care in the study area.

**Methods:**

A facility-based cross-sectional study was done from 1 March to 1 April 2019, among 612 randomly selected pregnant women attending antenatal care at Bako Tibe district public health facility. The data were collected via interview using a structured and pretested questionnaire. They were entered into EpiData Version 3.1 and SPSS Version 23 for cleaning and analyses. The variables, which were significant at *P* ≤ 0.2 in the bivariate logistic regression, were included in the multivariable analysis. The direction and strength of statistical association were measured by an odds ratio with 95% CI. A variable with a *P* value < 0.05 was considered a significantly associated factor with the outcome one.

**Results:**

In this study, the prevalence of unintended pregnancy was 33.3%, at 95% CI (29.8, 37.3). The factors that had significant association with unintended pregnancy were family size ≥ 6 (AOR = 8.0, 95% CI: 1.38–46.66), women who did not communicate about family planning with their husbands (AOR = 2.8, 95% CI: 1.50–5.20), and parity ≥ 5 (AOR = 3.0, 95% CI: 1.34–6.8).

**Conclusion:**

About one-third of the pregnant women reported that their pregnancy was unintended. Parity, family size, and lack of spousal communication showed a significant association with the problem. To decrease the current level of unintended pregnancy in the area, the Bako Tibe District Health Bureau and the health workers should work harder to scale up spousal communication on family planning.

## 1. Background

Unintended pregnancy is one that is either mistimed (unplanned) or unwanted totally. A woman is considered to have a mistimed pregnancy if she becomes pregnant at the time when she does not want to have a child [1].

Globally, out of the 213 million pregnancies, 80 million were unintended, and of them, 21.6 million ended by unsafe abortion, which claimed the lives of 47,000 women. Moreover, based on a Bayesian Hierarchical Model, the global trend of the magnitude of unintended pregnancy from 1990 to 2014 was estimated to be 44% [2]. In developing countries, out of 21 million pregnancies of women aged 15-19 years, unintended pregnancy accounts for around 49% [3]. An analysis of data from a study conducted in developing countries has indicated that the magnitude of unintended pregnancy in the countries varies from 13% to 82% [4].

Unintended pregnancy causes a major continuing social and health challenge in Africa, accounting for more than a quarter of pregnancies that occur annually in the region. It is a key risk factor for adverse pregnancy and maternal outcomes, including mortality and morbidity, associated with unsafe induced abortions [5, 6].

In Ethiopia, the wanted fertility rate is 3.6 children as compared with the actual total fertility rate, which is 4.6 children. This indicates that the women in Ethiopia are at the burden of having, on average, one child more than they want. According to the report of the Ethiopian Demographic Health Survey, the prevalence of unintended pregnancy in Ethiopia is 25%, of which 17% are unplanned and 8% are unwanted [7].

Women with unintended pregnancies have little attention to pregnancy-related complications and social support and few scores for self-care behaviors such as using folic acid or multivitamin of supplements, vaccination, and nutrition. These problems increase obstetric complications such as unfavorable pregnancy outcomes, maternal morbidity and mortality, premature birth, low birth weight, neonatal death, and infant abuse [3, 8].

Some studies revealed that children born from unintended pregnancy often suffer from lack of enough vaccination, malnutrition, poor breastfeeding, poor development and mental health, high rate of childhood morbidity and mortality, cognitive delay at 3 years old, more behavioral problems at 5 and 7 years old, and substance use at 14 years old than their counterparts [9, 10].

Addressing the factors contributing to unintended pregnancy is necessary to ensure the provision of safe and reliable service to reproductive age group women. Even though some studies have been done on the prevalence and associated factors of unintended pregnancy in Ethiopia, there is none in our study area. Therefore, this study is aimed at determining the magnitude and the factors associated with unintended pregnancy among pregnant mothers attending ANC at Bako Tibe district, West Shoa Zone, Oromia region, Ethiopia.

## 2. Methods and Materials

### 2.1. Study Period and Area

This study was conducted from March 1 to April 1, 2019, at Bako Tibe district health facilities. The study area is found in West Shoa Zone, Oromia Region, 251 km from Addis Ababa to the west. Its total population is 174,697. There are 4 health centers and one government hospital in the district.

### 2.2. Study Design and Population

A facility-based cross-sectional study was conducted from March 1 to April 1, 2019, among pregnant women who were receiving care at the ANC units in Bako Tibe district health facilities during the study. All the pregnant women who came for ANC service during the study were included, but the ones who were unable to communicate, for example, hearing impaired and seriously ill, were excluded from the study.

### 2.3. Sample Size

The total sample size used for this study was determined using a single population proportion formula (*n* = (*Zα*/2)^2^*pq*/*d*^2^). By considering the proportion of unintended pregnancy, which was taken from the study done in central Ethiopia, 36.4% [11], 95% confidence level, *z* values 1.96, a margin of error 4%, and 10% nonresponse, this resulted in 612 study participants. First, participants for each health facility were allocated using proportional to sample size. Then, the study participants were recruited using a simple random sampling technique.

### 2.4. Data Collection Tool and Procedure

Data were collected by using a structured and pretested questionnaire adapted from a study conducted on the same topic. Trained BSc midwives collected the data and supervised the collection process. To ensure the quality of the data, a two-day training was given to both the data collectors and the supervisors regarding the objective of the study, data collection tool, ways of data collection, checking the completeness of data collection tools, and how to maintain confidentiality. Before being entered into software, the data were properly coded, categorized, and checked for completeness, accuracy, clarity, and consistency by the principal investigator and supervisors. Furthermore, the data were double entered and compared to the original data. Simple frequencies and crosstabulation were done for missing values and variables.

### 2.5. Data Processing and Analysis

After the data had been checked for completeness and internal consistency, coded and double entered into EpiData Version 3.1 computer software package, and cleaned for inconsistency, they were exported to SPSS Version 23 for further data cleaning and analysis. Frequency and crosstabulation were conducted to check for any missing values. Descriptive statistics were used to present the prevalence of unintended pregnancy, and a binary logistic regression was used to see the association between the outcome variable and each independent variable. The variables that showed *P* ≤ 0.2 in the bivariate analysis were considered as a candidate for multivariable logistic regression analysis (using Enter Method); this was to control all possible confounders and to detect associated factors of unintended pregnancy. Hosmer and Lemeshow goodness-of-fit test (0.95) was used to assess whether the necessary assumptions were fulfilled. Multicollinearity test was carried out to see the correlation among the independent variables by using colinearity statistics (VIF). The direction and strength of statistical association were measured by an odds ratio with 95% CI. The adjusted odds ratio along with 95% CI was estimated to identify the associated factors for unintended pregnancy. Finally, statistical significance was declared at *P* value < 0.05.

## 3. Results

### 3.1. Sociodemographic Characteristics

In this study, 591 pregnant women were interviewed, making a response rate of 96.6%. The mean age of the study participants was 27.6 (SD ± 6.7), and 161 (27.2%) participants were within the age group of 25-29 years. Out of the 591 respondents, 306 (51.8%) were urban dwellers, 199 (33.7%) attended primary school, 346 (58.5%) were housewives, and 567 (95.9%) were married (Table 1).

### 3.2. Pregnancy- and Health-Related Characteristics

Few of the study subjects were with gravidity (137 (23.2%)) and parity (81 (17.6%)) greater than or equal to five. Of all the pregnant women, 77 (16.8%) got pregnant with less than two years from the previous birth, 385 (83.9%) had at least two children, and 316 (68.7%) had a son in their previous birth (Table 2).

### 3.3. The Magnitude of Unintended Pregnancy

**Figure 1:**
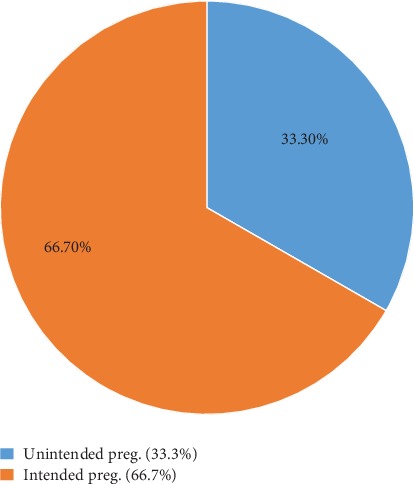
Magnitude of pregnancy among pregnant women attending ANC at Bako Tibe district, Ethiopia, 2019.

Out of the total study participants, 197 (33.3%) (95% CI (29.8-37.3)) responded that their pregnancy was unintended (Figure 1).

### 3.4. Awareness towards Contraceptive Method and Contraceptive Use

Almost all the respondents (560 (94.8%)) were aware of contraceptive methods; they also knew the types: 458 (81.8%) participants knew the injectable type of contraceptive method. For 452 (80.7%) respondents, health professionals were the main source of information (Table 3).

### 3.5. Factors Associated with Unintended Pregnancy

The relationship between each independent variable and the dependent variable was separately analyzed. In the bivariate analysis, the variables that were associated with unintended pregnancy were parity, family size, knowledge of ovulation time, having a son, spousal communication about family planning, decision-making to obtain health care, women who know about IUCD, history of unintended pregnancy, and awareness that unintended pregnancy is preventable.

The multivariate analysis was used to control confounding variables and to identify the factors associated with an unintended pregnancy. The pregnant women whose household family size was greater than 6 [AOR: 8, 95% CI (1.01, 46.6)] were 8 times more likely to report their pregnancy as unintended compared to those whose family size was less than or equal to two. The women who had parity of greater than 5 were 3 times more likely to report their pregnancy unintended [AOR = 3; 95% CI (1.34-6.8)] than those who had parity less than or equal to two (Table 4).

## 4. Discussion

In this study, the magnitude of unintended pregnancy among pregnant women attending antenatal care at Bako Tibe district was 33.3%, 95% CI (29.8, 37.3). The factors that were significantly associated with unintended pregnancy were family size, parity, having a son, and lack of spousal communication about family planning.

The prevalence of the unintended pregnancy observed in this study is in agreement with the ones found by studies conducted at Debre Markos (32.9%) and Addis Ababa (36.4%) [11–14]. But it is lower than the magnitudes reported from South Africa (64%), Nepal (54.5%), and Arsi zone (41%) [11–13]. This might be due to the difference in sample size, sociocultural characteristics, and health coverage of the study area, as well as the difference in the availability and accessibility of services (like access to modern contraceptives) for maternal health service in the countries. The extent of unintended pregnancy we found is, however, higher than the one reported for the national level (25%) [7] by the 2016 Ethiopian Demographic Health Survey. The difference might be due to the difference in the source population, where the present study focused only on pregnant women attending ANC at the health institution.

Like the result of a study conducted in Debre Markos Town, in our study, not using contraceptives was the main reason for the occurrence of unintended pregnancy (21.8%) [14]. This could be due to poor counseling during service delivery, which includes not introducing women with different types of contraceptive methods.

In this study, the family size was associated with an unintended pregnancy. The women with family size ≥ 6 were 8 times more likely to have unintended pregnancy compared to those with family size less than or equal to two. This finding is in agreement with the one reported by a study conducted in Felege Hiwot Hospital, Ethiopia, in which as family size increased, the rate of reporting pregnancy as unintended increased [15]. This might be the unmet need for family planning, and women could already have attained the number of families they desired.

In this study, parity was also significantly associated with an unintended pregnancy. Parity 5 and above women were more likely to experience unintended pregnancy than those who were with ≤2 parity. This finding is consistent with a finding at Gelemso Hospital, Ethiopia, in which the risk of unintended pregnancy increased with an increase in parity [16]. This is because high parity women might already have adequate children and practice sex for enjoyment rather than to have children. Also, it might imply the gaps in counseling and the provision of postpartum contraceptives.

Among the respondents who had children, those who had a son in their family were 3.9 times more likely to report their pregnancy as unintended compared to those who had no son in their family. Similarly, a study conducted in Pakistan showed that the women who had at least one son were almost three times more likely to report their recent pregnancies unintended compared to those who did not have a son [17]. Also, in a study done in Vietnam, increased number of live sons was positively associated with unintended pregnancy [6]. This implies that, culturally, male child preference is common in these countries, particularly in rural areas. The women who had a live male child may have been interested in preventing pregnancy for the sake of spacing or may no longer desire fertility, but with limited contraceptive use, they may experience an unintended pregnancy.

Furthermore, in this study, unintended pregnancy was associated with spousal communication about family planning. The women who did not communicate with their husbands were 2.8 times more likely to have unintended pregnancy compared to those who communicated. In a similar study done in Tigrai Region, Ethiopia, spousal communication has been found to be significantly associated with unintended pregnancy [18]. Also, a study conducted in Damon Gale District, Ethiopia, showed that women's perception that their husbands oppose family planning is one of the dominant factors for discouraging contraceptive practice in a wide variety of settings [19]. This implies that communication between couples regarding family planning helps women to use contraceptive methods to prevent unintended pregnancy.

### 4.1. Strength and Limitation of the Study

As a strength, the study considered cultural issues through involving female data collectors as it is common in the community to discuss the reproductive issue with the same gender. This also may result in reducing social desirability bias. However, the weakness of the study was that it was facility-based; it might not indicate the true rate of unintended pregnancy in the community, as many of the clients with unintended pregnancy are less likely to visit health institution. The responses might have been liable to social desirability bias. Since the sampling technique is nonprobability, sampling the finding is not generalizable to the study area.

## 5. Conclusion

One-third of the study participants reported that their pregnancy was unintended. The magnitude is high compared to the national average. Family size, parity, having a son, and lack of spousal communication on family planning were significantly associated with the unintended pregnancy among the pregnant women attending ANC at Bako Tibe district. Common reasons given by the respondents for not avoiding unintended pregnancy were family planning discontinuation, considering oneself as not fertile, and method failure. Health care workers of Bako Tibe district need to promote family planning services to minimize unintended pregnancy and to decrease parity and family size. Also, they need to give health education on the advantage of spousal communication on family planning, as it minimizes unintended pregnancy. The researcher also needs to conduct a qualitative study to explore additional factors.

## Figures and Tables

**Table 1:** Sociodemographic characteristics of respondents among pregnant women attending ANC at Bako Tibe district at public health centers and hospital, 2019 (*n* = 591).

Variable	Frequency (*n*)	Percent (%)
Age		
15-19	64	10.8
20-24	135	22.8
25-29	161	27.2
30-34	108	18.3
≥35	123	20.8
Residence		
Urban	306	51.8
Rural	285	48.2
Marital status		
Married	567	95.9
Single/divorced/widowed	24	4.1
Educational level		
Not able to read and write	202	34.2
Read and write	22	3.7
Primary school	199	33.7
High school	94	15.9
College and above	74	12.5
Occupation		
Government employee	63	10.7
Merchant	111	18.8
Student	18	3.0
Housewife	346	58.5
Daily laborer	44	7.4
Private work	9	1.5
Family size		
1-2	143	24.2
3-5	295	49.9
≥6	153	25.9

**Table 2:** Pregnancy- and health-related characteristics of pregnant women attending ANC in a public health facility in Bako Tibe district, Oromia, Ethiopia, 2019 (*n* = 591).

Variable	Frequency (*n*)	Percent (%)
Gravidity		
1-2	265	44.8
3-4	189	32
≥5	137	23.2
Parity		
≤2	246	53.6
3-4	132	28.8
≥5	81	17.6
Birth spacing in years		
Less than two years	77	16.8
≥2 years	381	83.2
Having a son		
Yes	316	68.7
No	144	31.3
Knowledge of ovulation time		
Know	175	29.6
Do not know	416	70.4
Reason for unintended pregnancy		
My husband wanted	19	9.6
Contraceptive method failure	34	17.2
I do not know about FP	3	1.5
I did not consider myself fertile	32	16.2
I discontinued FP	64	32.4
Short space between last birth	12	6.1
It was rape	3	1.5
I have no enough money to care	32	16.2

**Table 3:** Awareness towards contraceptive method of pregnant women attending ANC in a public health facility in Bako Tibe district, Oromia, Ethiopia, 2019 (*n* = 591).

Variable	Frequency (*n*)	Percent (%)
Awareness about FP		
Yes	560	80.7
No	31	19.3
Source of information about FP		
Radio	98	17.5
Television	237	42.3
Health professional	452	80.7
Awareness towards contraceptive method type		
Contraceptive pills	97	17.3
Injectable	458	77.5
Implants	363	64.8
IUCD	89	15.9
Spousal communication about FP		
Yes	327	58.4
No	233	41.6
Ever used contraceptive		
Yes	386	68.9
No	174	31.1
Type of contraceptive used		
Contraceptive pills	28	7.2
Injectable	282	72.7
Implants	105	27.1
IUCD	6	1.5
Awareness that UP is preventable		
Yes	134	76.1
No	426	23.9
Who decides for you to obtain health care		
My husband	157	26.6
Me and my husband	389	65
Me	47	7.9

**Table 4:** Multivariable analysis of factors associated with unintended pregnancy among women attending ANC in a public health facility in Bako Tibe district, Oromia, Ethiopia, 2019 (*n* = 591).

Variable	Pregnancy		COR (95% CI)	AOR (95% CI)	*P* value
	Unintended	Intended			
Family size					
≤2	20 (40%)	123 (86%)	1	1	
3-5	73 (24.7%)	222 (75.3%)	2.02 (1.17-3.47)	3.5 (0.63-19.9)	0.196
≥6	104 (68%)	49 (32%)	13.05 (7.2-23.30)	**8.0 (1.38-46.66)**	**0.020** ^∗^
Distance between home and health facility					
≤30 minutes	90 (31.7%)	194 (68.3%)	1	1	
One hour	64 (34.2%)	123 (65.8%)	1.12 (0.758-1.66)	0.40 (0.10-1.50)	0.192
One and a half hour	20 (29.9%)	47 (70.1%)	0.91 (0.514-1.63)	0.45 (0.11-1.81)	0.263
Two hours	12 (40%)	18 (60%)	1.43 (0.66-3.11)	0.31 (0.69-1.40)	0.128
≥2 hours	11 (47.8%)	12 (52.2%)	1.97 (0.84-4.64)	0.753 (0.15-3.75)	0.730
Knowledge of ovulation time					
Know	35 (20%)	140 (80%)	1	1	
Do not know	162 (38.9%)	254 (61.1%)	2.55 (1.67-3.88)	1.4 (0.72-2.72)	0.311
Parity					
≤2	54 (22%)	192 (78%)	1	1	
3-4	64 (48.5%)	68 (51.5%)	3.34 (2.12-5.27)	1.46 (0.81-2.65)	0.204
≥5	61 (75.3%)	20 (24.7%)	10.8 (6.02-19.53)	**3.0 (1.34-6.8)**	**0.007** ^∗^
Having a son					
Yes	158 (50%)	158 (50%)	5.8 (3.50-9.77)	**3.75 (1.94-7.22)**	**0.000** ^∗^
No	21 (14.6%)	123 (85.4%)	1	1	
Know about IUCD					
Yes	17 (19.1%)	72 (80.9%)	1	1	
No	173 (36.7%)	298 (63.3%)	2.45 (1.43-4.30)	2.22 (1.01-4.97)	0.046
Spousal communication about FP					
Yes	76 (23.2%)	251 (76.8%)	1	1	
No	114 (48.9%)	119 (51.3%)	3.16 (2.20-4.540)	**2.80 (1.50-5.20)**	**0.001** ^∗^
Awareness that UP is preventable					
Yes	28 (20.9%)	106 (79.1%)	1	1	
No	162 (38%)	264 (62%)	2.32 (1.46-3.6)	0.78 (0.38-1.60)	0.501
History of past unintended pregnancy					
Yes	34 (58.6%)	24 (41.4%)	3.21 (1.84-5.59)	**4.6 (2.1-10.46)**	**0.000** ^∗^
No	163 (30.6%)	370 (69.4%)	1	1	
The decision to obtain health care					
Husband	82 (52.2%)	75 (47.8%)	2.18 (1.09-4.37)	1.56 (0.58-4.16)	0.372
Me and my husband	100 (25.7%)	289 (74.3%)	0.69 (0.35-1.33)	0.36 (0.29-2.40)	0.893
Me	15 (33.3%)	30 (66.7%)	1	1	

Key: ^∗^variables associated at *P* < 0.05; AOR: adjusted odds ratio.

## Data Availability

The datasets used for analysis are available from the corresponding author on reasonable request.

## Abbreviations

ANC: Antenatal care.
